# Telomere Shortening and Its Association with Cell Dysfunction in Lung Diseases

**DOI:** 10.3390/ijms23010425

**Published:** 2021-12-31

**Authors:** Andy Ruiz, Julio Flores-Gonzalez, Ivette Buendia-Roldan, Leslie Chavez-Galan

**Affiliations:** Instituto Nacional de Enfermedades Respiratorias Ismael Cosío Villegas, Mexico City 14080, Mexico; andy.ruiz@iner.gob.mx (A.R.); juliofglez@gmail.com (J.F.-G.); ivettebu@yahoo.com.mx (I.B.-R.)

**Keywords:** telomere shortening, lung diseases, immune system, treatments

## Abstract

Telomeres are localized at the end of chromosomes to provide genome stability; however, the telomere length tends to be shortened with each cell division inducing a progressive telomere shortening (TS). In addition to age, other factors, such as exposure to pollutants, diet, stress, and disruptions in the shelterin protein complex or genes associated with telomerase induce TS. This phenomenon favors cellular senescence and genotoxic stress, which increases the risk of the development and progression of lung diseases such as idiopathic pulmonary fibrosis, chronic obstructive pulmonary disease, SARS-CoV-2 infection, and lung cancer. In an infectious environment, immune cells that exhibit TS are associated with severe lymphopenia and death, whereas in a noninfectious context, naïve T cells that exhibit TS are related to cancer progression and enhanced inflammatory processes. In this review, we discuss how TS modifies the function of the immune system cells, making them inefficient in maintaining homeostasis in the lung. Finally, we discuss the advances in drug and gene therapy for lung diseases where TS could be used as a target for future treatments.

## 1. Introduction

Telomeres are repetitive regions of noncoding DNA localized at the end of eukaryotic chromosomes to provide genome stability. The telomere length tends to be shortened with each cell division because of the normal process of DNA replication; thus, progressive telomere shortening (TS) occurs with age. Structurally, telomeres are nucleotide sequences comprising tandem repeats of TTAGGG attached to two protein complexes, shelterin, and telomerase, to regulate their attrition rate in each cell division [1,2,3,4]. In this regard, it has been reported that the telomerase, an enzyme multi-unit complex, has a catalytic component that is silent in normal cells, while it is reactivated in cancer cells to maintain TS and contributes to their indefinite proliferation to maintain tumor growth [5]. Thus, telomere length is a sign of cell fitness, but the attrition rate varies depending on cell type [6,7,8]. In the 1960s, Hayflick described the finite replicative capacity of somatic cells, providing the basis to clarify that TS is associated with the passage number and replicative capacity of cells [9].

In addition to age, other factors, such as exposure to inhalable particulate matter (PM 2.5 and 10), stress, smoking, obesity, sedentary lifestyle, poor diet, oxidative stress, DNA damage (caused by free radicals), and alterations in the shelterin complex or telomerase-associated genes promote continuous damage to cellular DNA to induce TS [10,11,12,13]. Currently, it is unclear how TS affects specific functions of immune system cells. In this context, we discuss the relationship between TS and alterations in the function of cells of the immune system as well as current advances in the development of drug and gene therapy as targets for future treatments of lung diseases.

## 2. New Lights on TS and Cell Senescence

During aging has been described diverse immunological alterations that alter the balance of immune cells as the accumulation of differentiated and less proliferative T cells; in this regard, cellular senescence is characterized by the development of time-dependent changes in global gene expression, epigenetic profile, and metabolism that end when a cell shows irreversible cell cycle arrest [14]. A cell under a senescence context has the ability to develop a specific phenotype called senescence-associated secretory phenotype (SASP), which is characterized by high expression of senescence-associated beta-galactosidase (SA-β-Gal), cyclin-dependent kinase inhibitor 2A (p16^INK4a^) and phosphorylated histone H2AX (γH2AX), all them as a marker of senescence cells [15]. Moreover, SASP increases the production and delivery of interleukins, inflammatory cytokines, and growth factors [16]. The SASP under an inflammatory context is a two-edged sword; it is helpful to stop cell division in response to stress or DNA damage. But, on the other hand, it can affect surrounding cells or neighboring tissues, promoting a tumoral effect [16,17]. In addition, the cell senescence phenotypes offer irreversible DNA alterations due to modifications in the DNA methylation. Thus, the cell stress induced by oxidative stress, replicative stress, genotoxic agents, mitochondrial dysfunction, irradiation, as well as inflammatory stress by the cytokines storm and telomere shortening are other inducers of premature cellular senescence [18].

Recent studies have demonstrated that some therapies as those based on chemotherapy and radiation, which are used in cancer, act as inducers of senescent cells [19]. Thus, the Therapy-Induced Senescence induces genomic damage or epigenetic modifications that development a senescence-like terminal proliferation arrest [20]. Therefore, cellular senescence is a topic of interest in the study of mechanisms associated with aging, but also this topic has a growing body of evidence about its role in pathological processes, including the search for chemotherapeutic agents. In this context, senolytic treatments are an effective therapeutic intervention used to eliminate senescent cells in patients at risk of pathological processes [21]. Some senolytic drugs are inhibitors of the anti-apoptotic proteins BCL-2, BCL-xL and BCL-w, as the navitoclax [22], metformin that reduce senescent markers [23], natural compounds which are called flavones and they exhibit prooxidant activity [24], and those that modulate the autophagy process as azithromycin and roxithromycin [25].

In addition, other factors such as lifestyle and nutrition play a role in inducing a phenomenon called immunosenescence, where the proper function of the immune cells is compromised. Current evidence suggests the immunosenescence is not only a mechanism aging-related and increased type-1 interferon signaling [26]. Infection-related processes, such as multidrug-resistant tuberculosis (MDR-TB), have been also associated with a reduced telomere length (TL), increasing mitochondrial DNA copy number [27]. A recent report indicated that individuals over 50 years of age present interstitial lung abnormalities, and although they are asymptomatic respiratory individuals, they have increased serum levels of matrix metalloproteinases (MMP)-1, 7, 13 and interleukin (IL)-6 compared to older control patients [28]. Thus, it is confirmed that factors as aging and therapy-induced senescence could be associated with the development of pulmonary anomalies. Although they can be unnoticed, with aging, they are still latent and may increase the risk of several pathologies, moreover these alterations, we cannot forget infectious agents.

Currently, cell senescence is considered an important player in diseases such as COVID-19 where the senescence landscape is present [29,30]; idiopathic pulmonary fibrosis (IPF) for which has been observed an increase in factors such as β-galactosidase, p21, p16, p53 and high levels of plasminogen activator inhibitor 1 (PAI-1) associated with alveolar type 2 cells [31,32,33]; chronic obstructive pulmonary disease (COPD) that links to mTOR activation to favor the development of lung emphysema, pulmonary hypertension, and inflammation [34].

Previous studies showed the possibility that premature senescence may significantly modify the circadian rhythms, suggesting one explication to the susceptibility to other diseases [35]. For example, aged individuals show low expression of ACE2 and have distinct diurnal changes associated with the rhythmic expression of Per2, one key repressor that constitutes the feedback loop of mammalian clock circuitry [36,37]. CE2 controls the proinflammatory microenvironment regulating angiotensin II (ATII) and angiotensin 1–7 levels. Under these conditions, aged individuals are less susceptible to the SARS-CoV-2 infection, but frequently, they present severe disease outcomes [38]. Patients with IPF have been related to high clock protein REVERBα expressions, one phenomenon that we have limited understanding of [39].

To better understand the role of cell senescence in the development of pathologies, new models of premature cell senescence based on the deletion of genes involved in the regulation of the cell cycle have been developed [40]. On the other hand, lysosomal activity, expression of Ki67, RPS6, and beta-galactosidase, and soluble molecules related to the SASP have been explored as biomarkers to discriminate senescent subtypes [41,42,43]. Moreover, these molecules are identified at a different cellular level, from the genetic to protein level, and they provide information regarding signaling pathways of senescence [44,45]. New methodologies allow us to analyze these molecules better; for instance, the combination of flow cytometry with high-content image analysis has better detailed the positive correlation between leukocyte cellular senescence and aging [46]. Quantification of S-nitrosoglutathione reductase (GSNOR) levels has been proposed as a predictor of senescence. The redox-based posttranslational modification S-nitrosylation regulates cellular homeostasis broadly, including metabolic, cardiovascular, and immune function, and GSNOR levels decrease with aging resulting in mitochondrial damaged [47].

Analyzing one of the most important contributions to senescence, the TS, it has been explored that telomere length is a biomarker of cellular aging-related to drugs therapies [48], diseases [49,50]. Moreover, it has been associated with a broad spectrum of cellular inflammatory functions named “inflammaging” [51]. This phenomenon was detailed by Fraceschi et al. [52], as a progressive increase in low-grade inflammation by a continuous antigenic load and stress. However, under a chronic disease condition, there is an accelerated inflammatory process by an accelerated aging process [53]. In this last point, it has been described that some chronic diseases are related to a circulating immune cell landscape, which is characterized by the increase in natural killer cells, age-associated B cells, inflammatory monocytes and age-associated dendritic cells [29,54], TS and lower soluble factors such as vascular endothelial growth factor A (VEGF-A) [55]. Furthermore, regulators of telomere length can be subject to regulation by NF-kB signaling [56], and telomere activity can be modulated via ROS-mediated exacerbation or exposure to inflammatory molecules such as IL-6 or TNF [57].

**Figure 1:**
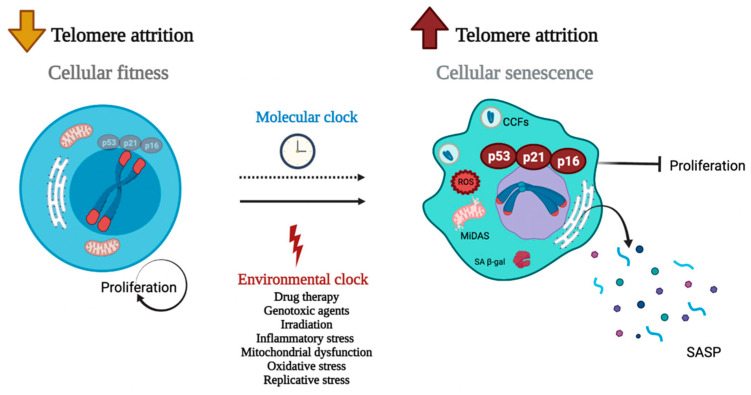
Telomere dysfunction regulates cellular senescence. All somatic cells have a molecular clock that leads them to develop a senescence phenotype (dotted line). This process leads to a gradual and controlled loss of telomeres so that under stimulation, cells can respond appropriately (cell fitness). However, this process can be accelerated (solid line) under environmental clock conditions given by drug therapy, genotoxic stress, irradiation, inflammatory stress, mitochondrial dysfunction, oxidative stress, or replicative stress. The environmental clock is associated with an accelerated rate of telomere decay, leading cells to acquire senescence-associated secretory phenotype (SASP). Some cellular markers of the cellular senescence process are mitochondrial dysfunction-associated senescence (MiDAS), cytoplasmic chromatin fragments (CCFs), B-galactosidase production, and regulatory factors such as p53, p21, and p16. During the last years, the SASP profile given by the uncontrolled secretion of cytokines such as IL-6, IL-8, TNF, IFN has been studied to classify and identify cells in senescence induced by telomere attrition.

Although there is an apparent relationship between senescence-age-telomeres, further studies are needed to better understand these complex interactions in human cells and tissues. Consistent with this view, the protein called protection of telomeres 1 (POT1), a component of the shelterin complex, acts as a regulator of telomeres and which expression is related to senescence [58,59]. In mice, POT1a represses the activity of the DNA damage machinery and regulates negatively the telomere length, while POT1b promotes telomerase recruitment to telomere elongation [60,61,62]. However, it is not clear what the expression of one subunit or the other depends on. In summary, we show the main differences between a young and a senescent cell in Figure 1.

## 3. TS and Lung Disease Development

With aging, the immune cells display TS, which affects the efficacy of activating effector mechanisms, such as cytotoxicity, phagocytosis, and cytokine delivery. This phenomenon is known as immunosenescence, and it is one of the mechanisms more described that plays a significant role in lung diseases such as IPF, familial pulmonary fibrosis (FPF), and lung cancer [63,64,65].

Mutations in proteins associated with shelterin and telomerase complexes decrease telomerase activity and are related to TS [66,67,68]. Reports suggest that mutations in POT1 and telomere protection protein 1/adrenocortical dysplasia protein (TPP1/ACD), members of the shelterin complex, are associated with TS [69]. Similarly, mutations in TERT, TERC, DKC1, PARN, RTEL1, TINF1, OBFC1, and NAF1, which are regulatory proteins in the telomerase complex, contribute to TS and cellular senescence by reducing telomerase activity [70,71]. These mutations are associated with hereditary syndromes such as Von Hippel–Lindau disease and dyskeratosis congenital syndrome. In both diseases, there is a relationship between TS and the development of lung diseases of transgenerational inheritance [72,73]. TS is described in 25% of patients with IPF and >50% of patients with FPF [74]. Moreover, it increases epithelial cell apoptosis; in particular, FPF patients exhibiting TS have a worse prognosis and high morbidity after lung transplantation than those without TS [64,75]. Different lung diseases are associated with mutations in both the shelterin and telomerase complexes. Table 1 summarizes the main lung diseases associated with mutations in these complexes.

Recently, a family of long noncoding RNAs named telomeric repeat-containing RNAs (TERRA) critical for the recognition of shelterin and telomerase complexes to maintain telomere stability has been reported [76,77,78,79]. TERRA is delivered through exosomes and also exists as a cell-free form to promote cell communication and activate the innate immune response by stimulating the secretion of tumor necrosis factor (TNF), interleukin (IL)-6, and C-X-C chemokine 10 (CXCL10) [80]. Thus, TERRA could be a helpful tool to induce a specific inflammatory microenvironment during respiratory diseases, primarily those where there is a switch between pro and anti-inflammatory environment as in cancer.

## 4. Influence of Immune Cells with Shorter Telomeres on Development of Lung Diseases

TS occurs in response to a dynamic environment and age, and it is involved in the induction of immune cells plasticity, this means, cells can modulate their functions in concordance with the microenvironment [81,82,83]. For instance, a previous study reported that the lymphocytes (CD8+ and B cells) of people producing higher antibody titers on influenza vaccine administration exhibited longer telomeres [81]. Another study proposed that leukocytes exhibiting TS could be a biomarker of liver injury caused by excessive production of reactive oxygen species induced by antituberculosis drugs, such as rifampicin and isoniazid [84]. Furthermore, a recent clinical study indicated that TS is associated with cardiovascular disease risk in populations with high stress and cotinine levels due to smoking [85]. Collectively, these findings show that telomere length is associated with the ability of the immune system to respond to a dynamic environment because the integrity of telomeres is essential for the integrity of chromosomes.

It is well established that inflammation is a common pathological factor in several diseases; for example, high IL-18 and IL-1β levels are associated with an increased risk of cardiovascular diseases because these cytokines increase local inflammation, oxidative stress, and procoagulant mediator production and impair vasodilation [86]. Reports indicate that the interaction between low-density lipoprotein (LDL) and free fatty acids (serum albumin-bound palmitic acid) triggers IL-1β production in macrophages via an oxidized low-density lipoprotein receptor 1 (LOX-1)-dependent pathway [87]. This is relevant in the context of TS because Wang et al. [88] have demonstrated that LDL uptake via LOX-1 promotes mitochondrial damage and decreases telomerase activity, inducing a senescence phenotype through p21 upregulation by TP53. We speculate that similar mechanisms could be involved in TS during lung diseases as lung diseases of both noninfectious and infectious origin exhibit increased IL-1β levels and LOX-1-dependent acute inflammation [89,90].

Findings explaining the signaling mechanism in the inflammation/TS axis are limited; however, it is now clear that there is a link between these phenomena. Inflammation causes an imbalance between reactive oxygen species (ROS) production and antioxidant ability named oxidative stress. In this context, ROS could induce discontinuities in one strand of the DNA double helix named singles-strand breaks (SSBs) at telomeric regions. This phenomenon causes collapsed replication forks, causing an accumulation of unreplicated ssDNA and finally manifesting as TS [91,92]. In addition, the TS could be caused by a sensitivity of site-specific DNA damage at 5′-GGG-3′ sequence in telomere sequence to oxidative stress [93,94]. In this regard, Kang et al. [95] observed that macrophages with TS exhibited mitochondrial abnormalities, oxidative stress and caused lung hyperinflammation on infection with *Staphylococcus*, shedding light on age-related pathologies, including lung diseases. In autoimmune diseases, a positive association between TS and the shared epitope (SE), a five amino acid sequence motif in residues 70–74 of the HLA-DRβ chain has been established; interestingly, SE is associated with excessive inflammation [96]. Although the immunological consequences remain unexplored, human studies and mice models suggest that chronic inflammation increases TS [56,97].

T cell maturation affects telomere length; from the naïve status to central memory and effector memory, there is a progressive decline in the expression of a member of the telomerase complex, the human telomerase reverse transcriptase (hTERT), which induces TS to limit uncontrolled or unnecessary T cell clonal expansion [98]. Similarly, telomerase-null mice experience more TS than wild-type mice; consequently, they exhibit decreased T cell development (CD4+ and CD8+) due to intrinsic apoptosis pathway upregulation and high PD-1 expression [99]. Thus, it is likely that T cells with TS undergo early apoptosis in the hematopoietic reserves; consequently, intrathymic precursor death is increased, and T cell development is decreased.

Yang et al. [63] demonstrated that the telomeres of naïve T cells from lung cancer patients were shorter than those of naïve T cells from healthy donors and that this was associated with advanced clinical stage. Conversely, in allergic diseases, the presence of shortened telomeres appears to induce airway inflammation; a positive correlation between leukocytes with shortened telomeres and inflammation in patients with severe asthma has been reported [100]. Piñeiro-Hermida et al. [101] found that mice with increased TS rates due to genetic deficiencies (*G3 Tert*−/− mice) or induced by 6-thio-2′-deoxyguanosine administration exhibited eosinophilia and low circulating IgE levels and that TS affects the differentiation of club cells, which are essential for eliminating harmful substances inhaled into the lungs. Tung et al. [102] proposed that snoring accelerates TS in atopic patients, primarily in those with asthma and allergic rhinitis; however, a limitation of this study was that it did not identify which immune cell population is more sensitive to increased TS. Moreover, an association has been found between the single nucleotide polymorphism *MUC5B* rs35705950 related to IPF predisposition and telomere shortening in leukocytes from two different cohorts. However, further studies elucidating the direct role of this polymorphism with telomere biology are lacking [103]. Moreover, the process of fetal programming of the telomere system raises the possibility that telomeropathies are the product of genetic changes in cells during embryogenesis due to exposure to air pollution [104]. Investigative evidence suggests that early fetal exposure to air pollution and cigarette smoke during both the second and third trimesters of pregnancy affects telomere length in newborns [105,106].

Regarding cytotoxic T cells, experimental evidence indicates that CD8+CD28+ T cell count can be used as a predictive value of negative anti-tumoral response in patients with lung metastases from non-small cell lung cancer, which means, a low count is associated with a negative response to treatment [107]. In fact, in lung cancer with metastasis, it has been reported that higher levels of CD8+CD28+ T cells predicted favorable overall survival [108,109].

It is important to note that CD28+ T cell subpopulations represent a subpopulation of memory T cells that reconstitute the spectrum of effector T cell subsets, and these cells participate in the inflammation process [65]. Furthermore, observations suggest that CD28+ cells can activate telomerase and maintain telomere length during T lymphocyte stimulation [110]. It is likely that these cytotoxic cells are involved in the high morbidity of fibrotic interstitial lung diseases in patients with telomere dysfunction after a lung transplant. It seems that the status of CD28+ T cells behaves as a double-edged sword: an increase in CD28+ cells is tumorigenic in the early stage but related to more prolonged survival, and loss of CD28 is related to metastasis [111,112]. In contrast, a consequence of accumulating CD8+/CD28− T cells is that they can lose their function, have reduced expression of effector molecules (granzyme B and perforin), and reduced cytotoxic T-lymphocyte (CTL) activity [113]. However, further studies are needed to clarify whether a shortened telomere affects the capacity of cytotoxic cells to eliminate altered cells, such as those involved in fibrosis development.

Alveolar epithelial type II cells (AT2) are another type of cells associated with TS; they are involved in the development of pulmonary fibrosis and display a senescence-associated secretory phenotype; TS in AT2 is associated with alterations in telomere shelterin, specifically in the TRF1 protein [114,115]. Naikawadi et al. [114] observed that lung remodeling is characterized by increased AT2 cells, accumulation of senescence-associated β-galactosidase+ epithelial cells, and collagen-expressing cells however, only AT2 cells had TS. Another report mentioned that AT2 cells from IPF patients with mutations in telomerase reverse transcriptase (TERT-PF) display an increase in the phosphorylation of H2A histone family member X (γH2AX), which initiates the DNA damage response [116]. A telomerase activation such as a treatment, Bär et al. [117], testing this phenomenon using adenovirus vector gene therapy to carry the telomerase *Tert* gene in a mouse model of aplastic anemia due to TS (Tert-deficient mice model) observed an increase in telomere length in peripheral blood and bone marrow cells.

In contrast, Waisberg et al. [118] suggested that AT2 cells from IPF patients have abnormal regulation of telomerase, which increases apoptosis and consequently affects the regenerative capacity of alveolar epithelial cells. Furthermore, a high TS rate in IPF patients <60 years old is considered a poor prognosis because these patients present with more nonspecific hematological and/or immunological abnormalities [64]. Although there is limited information regarding the role of circulating microRNAs in the induction of TS and its link with the regulation of senescence in lung diseases, a recent report suggests that the upregulation of circulating microRNAs is associated with the regulation of critical signaling pathways, including senescence, which is in agreement with a previous report that indicated that miR-34a regulates c-Myc and FoxM1; interestingly, both molecules have a role in hTERT transcription [119,120].

Previous reports suggest that TERT deficiency in AT2 cells promotes susceptibility to senescence, inflammation, and fibrosis in the lungs [121]. In fact, reports suggest that hTERT has a protective role against fibrosis [122]. In an IPF model, Naikawadi et al. [114] demonstrated that TS in AT2 leads to spontaneous development of lung fibrosis due to high levels of the regulatory cytokine TGF-β1; in addition, increased lung microbiome dysbiosis is observed. Notably, IPF patients are highly susceptible to infections with bacteria such as *Staphylococcus* and *Streptococcus* [123,124], and microbiome dysbiosis probably plays a major role in favoring this high susceptibility.

It has also been shown that patients who have undergone allogeneic hematopoietic stem cell transplantation reverse the cellular phenotype of exhaustion, spontaneous apoptosis, and senescence [125]. Borie et al. [126] demonstrated that AT2 cells, alveolar macrophages, and lymphocytes from patients with interstitial lung disease exhibit mutations in the regulators of telomere length 1 (RTEL1), which favors TS. Awad et al. [127] proposed a model in which low RTEL1 levels were associated with immunological abnormalities in CD34+ cells, B cells, and T cells. In consonance, previously has been reported that dyskeratosis congenita patients exhibit a hypomorphic mutation in *RTEL1* as the underlying basis of the clinical and cellular phenotypes [128]. In addition, it has been demonstrated that alveolar macrophages with shortened telomeres from COPD patients exhibit mitochondrial damage, which induces oxidative stress; hyperactivation of the inflammasome; and elevated levels of proinflammatory cytokines such as IL-1β, TNF, IL-6, IL-8, monocyte chemotactic protein (MCP)-1, Hu-GRO, and intercellular adhesion molecule (sICAM)-1, which together perpetuate pulmonary inflammation during COPD [95,129,130].

Previous reports suggest that TS in COPD is caused by inflammation and oxidative stress induced by the inhalation of external factors such as nitrogen dioxide, ozone, gasoline, diesel exhaust, and tobacco smoke, which disturb the oxidant/antioxidant balance [131,132]. Interestingly, Tanveer Ahmad et al. [133] demonstrated that TPP1 decreases in COPD patients and that the TPP1-Sirtuin 1 (Sirt1) interaction is altered in smokers. Consequently, the TPP1 level is decreased, which increases telomeric DNA damage and cellular senescence.

In parallel, some studies suggest that TS affects the regulation of the immune response. It has been proposed that severe acute respiratory syndrome coronavirus 2 (SARS-CoV-2) infection in older persons is associated with T-cell lymphopenia because TS depends on age; in this regard, recently was confirmed that old adults with SARS-CoV-2 infection have compromised the telomere length-dependent T-cell proliferative response, contributing to the profound T-cell lymphopenia among old adults [134]. In consonance with the previous statement, reports suggest that the high morbidity of patients with SARS-CoV-2 infection and comorbidities (cardiovascular and lung diseases) is attributable to the limited activation and proliferation of T cells [135]. Overall, findings to date indicate that TS is a phenomenon associated with the severity of SARS-CoV-2 infection [136]. It was related to decreased levels of sirtuins, a family of nicotinamide adenine dinucleotide (NAD+)-dependent enzymes that regulate energy metabolism, mitochondrial function, biosynthesis, gene expression, calcium signaling, immunological functions, and aging [137]. Another axis affected by TS is the interferon-stimulated gene 15 (*ISG15*); its upregulation is related to chronic inflammatory states associated with aging [138]. Recently, it has been shown that SARS-CoV-2 infection can antagonize *ISG15* activation through direct glycosylation (de-ISGylation) mediated by the papain-like protease of SARS-CoV-2, regulating the antiviral response of *ISG15* and the melanoma differentiation-associated protein 5 (MDA5), which is the responsible for the upregulation of interferon-stimulated genes [139].

**Figure 2:**
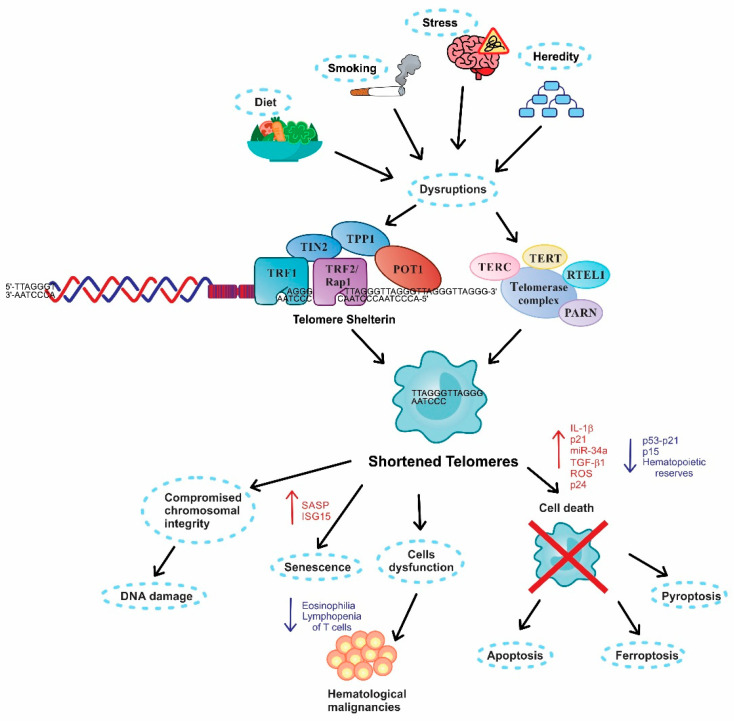
Schematic of accelerated telomere shortening—causes and its biological consequences. Insults by environmental or genetic factors induce disruptions in one of two essential complexes that regulate the telomere length: (1) telomere/shelterin, integrated with TRF1 (telomeric repeat factor 1), TRF2 (telomeric repeat factor (2), RAP1 (repressor/activator protein (1), TIN2 (TRF1-interacting nuclear factor (2), TPP1 (tripeptidyl peptidase (1), and POT1 (protection of telomeres 1) and (2) telomerase complex, composed by TERC (telomerase RNA component), TERT (telomerase reverse transcriptase), RTEL1 (regulator of telomere elongation), and PARN (gene encoding poly(A)-specific ribonuclease) proteins. These disruptions induce rapid telomere shortening (TS) (upper panel). Consequently, TS promotes the loss of chromosomal integrity and DNA damage, enabling an inflammatory microenvironment disbalance characterized by high levels of IL-1b, ROS, and TGF-b1 (lower panel). This figure was done with CorelDRAW Graphics software.

The association between telomere length and pulmonary disorder development requires further investigation. As previously discussed, telomeres are susceptible to insults by environmental or genetic factors leading to TS development through disruptions in both the telomere/shelterin or telomerase complex (Figure 2, upper panel). Immune cells are not excluded from TS, and when there is a development of pulmonary disorders, both infectious and noninfectious, because diverse immune functions are altered. This knowledge raises the possibility that TS participates in regulating the inflammatory process, perpetuating or discontinuing diverse immune cell phenotypes (Figure 2, lower panel). Currently, there is a need to establish new treatments and diagnostic tests for pulmonary diseases. TS in immunological cells has been discussed as a potential tool for designing better testing and therapies, distinguishing the physiopathology spectrum of diverse lung diseases, and improving patients’ quality of life.

## 5. Immunotherapy, Pharmacology, and Genetic Therapy

Up to one-third of people with FPF have TS and carry a telomere-related mutation; regardless of the IPF phenotype, individuals with TS and telomere-related mutations have more rapid disease progression [140,141,142]. In fact, reports show that TS is associated with susceptibility to IPF [75,143,144]. Recently, it has been reported that the use of both antifibrotic drugs nintedanib and pirfenidone reduced the decline in forced vital capacity in IPF patients with telomere-related gene mutations [145,146].

In patients with interstitial lung disease and non-IPF associated with short telomeres, the use of a synthetic steroid derived from ethinyltestosterone named danazol has been reported to lengthen telomeres, which improves both the radiological appearance and lung function tests [147,148,149]. However, studies on congenital dyskeratosis have not found a benefit of androgen therapy on telomere length, suggesting that the effect of androgens on hematological outcomes may be independent of telomere length [150]. Furthermore, the long-term administration of these agents may be associated with liver toxicity, and there are reports of worsening pulmonary fibrosis after danazol initiation and withdrawal [142].

To date, there are some drugs in clinical trials such as Nandrolone or Danazol that various investigations are being carried out regarding drugs that can inhibit telomeric dysfunction, by inducing telomerase activation [142,151]. In vitro data show that calcineurin inhibitors, particularly cyclosporin, can shorten telomeres more significantly than rapamycin [147]. Although the impact of this possible accelerated shortening on the bone marrow reserve is unknown, a trial target of rapamycin inhibitors in mammals in recipients with persistent cytopenia could be considered to allow for lower doses of calcineurin inhibitors.

CD8+ T lymphocytes from human donors infected with human immunodeficiency virus and treated with CAG showed increased telomerase activity, a moderate delay in telomere attrition, and an increase in the proliferation potential of CD8+ T lymphocytes and production of cytokines and chemokines, increasing the antiviral potential [152]. CAG-induced increases in telomerase activity are blocked by mitogen-activated protein kinase (MAPK) and extracellular signal-regulated kinase (ERK) inhibitors; therefore, it is likely that the enhanced antiviral functions associated with CAG are mediated through the ERK/MAPK pathway [152,153]. However, CAG is an effective telomerase activator, increasing telomerase activity and human CD4 and CD8 T cell proliferative potential and, CAG has been shown to enhance the antiviral ability of cells. Therefore, it can improve acquired immunodeficiency syndrome. However, further studies are required to demonstrate its effectiveness in lung diseases.

Table 1 presents a summary of the current treatment for lung diseases associated with mutations in both shelterin and telomerase complex inducing TS; however, there is a lack of clinical trials for several diseases, highlighting those future studies must focus on the development of drugs to target these mutations.

## 6. Conclusions

TS has a negative impact on the adequate activation of circulating immune cells, the gradual attrition rate of telomeres is related to immunosenescence and probably plays an important role in the progression of different pulmonary pathologies by mutations in telomere protein complexes (telomeropathies). Recent research has shown that TS can also be caused by an infectious origin, where the senescence phenotypes of immune cells perpetuate, and it is characterized by the increased frequency of memory T cells and a loss of naïve T cells, which opens a new panorama in therapies focused on the activation of telomerase function in the affected tissue, as in the case of the current SARS-CoV-2 pandemic, IPF, COPD, and lung cancer. TS is a key process with high potential to be used as a target to modulate lung diseases where a high TS rate is fundamental for the outcome; for instance, it is necessary to clarify the specific cellular subpopulations responding first to the damage stimuli generated by shortened telomere length, or if the inflammation is a consequence of TS or vice versa.

**Table 1:** Lung diseases associated with shortened telomeres: Related mutations and treatment.

Telomeropathies	Gene, Protein Name (S) Related	Treatment	Reference
Lung cancer	TERT, TERC, *PARN*, *TINF2*, NAF1, DKC1, RTEL1	Rapamycin	[154]
		Prednisone Cyclosporine Cyclophosphamide Dasatinib Quercetin Cycloastragenol (CAG) Danazol	[107,142,147]
IPF		Nintedanib Pirfenidone Alemtuzumab GRN510	[121,133,145,152,155,156]
Dyskeratosis congenita	TERT, TERC, DKC1, or *TINF2*	Danazol Oxymetholon Nandrolone Cycloastragenol CAG) Etoposide	[142,147,150]
Acute interstitial pneumonia Cryptogenic organizing pneumonia Smoking related interstitial lung disease	TERT	NS	[157] [140] [158]
Pleuroparenchymal fibroelastosis Hypersensitivity pneumonitis	TERT, TERC, RTEL1	NS NS	[141] [159]

DKC1, dyskerin pseudouridine synthase 1; NAF1, nuclear assembly factor 1; PARN, gene encoding poly(A)-specific ribonuclease, TERC; telomerase RNA component, TERT; telomerase reverse transcriptase, TINF2; gene codified TRF1-interacting nuclear factor, RTEL1; regulator of telomere elongation, NS; nonspecific.
